# Dallas Medical and Surgical Society

**Published:** 1914-12

**Authors:** 


DALLAS MEDICAL AND SURGICAL SOCIETY.—The feature of the bimonthly meeting of the Dallas Medical and Surgical Society, which was held October 7th at the home of Dr. and Mrs. J. W. Bourland, Oak Lawn and Lemmon Avenues, was the demonstration of the pulmotor, lungmotor and self-rescuer by Dr. John R. Worley. The pulmotor and lungmotor are instruments for restoring asphyxiated and half-drowned persons, and the self-rescuer is an apparatus for enabling firemen to enter burning buildings to rescue the inmates. The self-rescuer, it is said contains oxygen enough to sustain life thirty minutes.

Chief Magee and Capt. John Redman of the fire department, and Fire Marshal J. W. Thompson, who were the guests of the society, witnessed the demonstration.

Reports of cases were made at the meeting by Dr. Edwin J. Reeves, Dr. J. B. Smoot and Dr. Alfred I. Folsom. Dr. Spencer J. Davis read a paper on "The Relation of Nose and Throat to Immunity and Disease."

The meeting was the first one of the season, and the attendance was unusually large. After the program refreshments were served by Mrs. Bourland.

### A Strenuous Test.

A muscular Irishman strolled into the examination room where candidates for the police force are put to a physical test.

"Strip," ordered the Police Sergeant.

"What's that?" demanded the unitiated.

"Get your clothes off, and be quick about it," said the doctor.

The Irishman disrobed and permitted the doctor to measure his chest and legs, and to pound his back.

"Hop over this bar," ordered the doctor.

The man did his best, landing on his back.

"Now, double up your knees and touch the floor with your hands."

He sprawled face downward on the floor. He was indignant, but silent.

"Jump under this cold shower," ordered the doctor.

"Sure, that's funny," muttered the applicant.

"Now run around the room ten times to test your heart and wind," directed the doctor.

The candidate rebelled. "Oi'll not. I'll stay single."

"Single?" asked the doctor, surprised.

"Sure," said the Irishman, "what's all this fussing got to do with a marriage license?"

He had strayed into the wrong office.

### Inside Information.

Various doctors, among them many specialists, were called as witnesses in a case in a San Francisco court, with a view to ascertaining what killed a woman whose death was in question in an insurance litigation.

They all testified they had examined the woman professionally, and the consensus of opinion was that the dead woman had suffered from an affection of the liver which caused that organ to shrink materially.

The last doctor on the witness stand was a young hospital interne. He testified that instead of a shrunken liver the dead woman had an abnormally large liver.

Do you mean to sit there on the stand and swear that this woman had an enlarged liver when all these eminent authorities have sworn that her liver was wasted and shrunken?"

"I do," replied the young doctor.

"How comes it you set yourself up against these eminent practitioners—you, a young squirt of a doctor, with no practice, and only a few months out of a medical school? How do you know this woman had an enlarged liver?" thundered the lawyer.

"I performed the autopsy," answered the young doctor.—*Victoria Fact,* November 6, 1914.

